# Assessment of Surgical Accuracy in Maxillomandibular Advancement Surgery for Obstructive Sleep Apnea: A Preliminary Analysis

**DOI:** 10.3390/jpm13101517

**Published:** 2023-10-22

**Authors:** Jean-Pierre T. F. Ho, Ning Zhou, Tom C. T. van Riet, Ruud Schreurs, Alfred G. Becking, Jan de Lange

**Affiliations:** 1 Department of Oral and Maxillofacial Surgery, Amsterdam UMC, University of Amsterdam, Meibergdreef 9, 1105 AZ Amsterdam, The Netherlands; n.zhou@amsterdamumc.nl (N.Z.); t.c.vanriet@amsterdamumc.nl (T.C.T.v.R.); r.schreurs@amsterdamumc.nl (R.S.); j.delange@amsterdamumc.nl (J.d.L.); 2 Academic Centre for Dentistry of Amsterdam (ACTA), University of Amsterdam and Vrije Universiteit Amsterdam, 1081 LA Amsterdam, The Netherlands; 3 Department of Oral and Maxillofacial Surgery, Northwest Clinics, Wilhelminalaan 12, 1815 JD Alkmaar, The Netherlands

**Keywords:** obstructive sleep apnea, surgery, orthognathic surgical procedures, osteotomy, accuracy

## Abstract

This retrospective study aimed to: (1) investigate the surgical accuracy of maxillomandibular advancement (MMA) in obstructive sleep apnea (OSA) patients, with a specific focus on maxillary and mandibular advancement and counter-clockwise rotation and (2) investigate the correlation between the amount of achieved advancement and the reduction in the relative apnea hypopnea index (AHI). Sixteen patients, for whom a three-dimensional virtual surgical plan was generated preoperatively and a computed tomography scan (CT) or cone-beam computer tomography (CBCT) was acquired postoperatively, were included. The postoperative CT or CBCT was compared to the virtual surgical plan, and differences in the mandibular and maxillary advancement and counter-clockwise rotation were assessed. Maxillary and mandibular advancement (median 3.1 mm, *p* = 0.002 and 2.3 mm, *p* = 0.03, respectively) and counter-clockwise rotation (median 3.7°, *p* = 0.006 and 4.7°, *p* = 0.001, respectively) were notably less than intended. A significant correlation was found between the planned maxillary advancement and the difference between the planned and actual maxillary advancement (*p* = 0.048; adjusted R^2^ = 0.1979) and also between the planned counter-clockwise rotation and the difference between the planned and actual counter-clockwise rotation for the mandible (*p* = 0.012; adjusted R^2^ = 0.3261). Neither the maxilla-first nor the mandible-first surgical sequence proved to be superior in terms of the ability to achieve the intended movements (*p* > 0.45). Despite a significant reduction (*p* = 0.001) in the apnea hypopnea index (AHI) from a median of 62.6 events/h to 19.4 events/h following MMA, no relationship was found between the extent of maxillary or mandibular advancement and AHI improvement in this small cohort (*p* = 0.389 and *p* = 0.387, respectively). This study underlines the necessity for surgeons and future research projects to be aware of surgical inaccuracies in MMA procedures for OSA patients. Additionally, further research is required to investigate if sufficient advancement is an important factor associated with MMA treatment outcome.

## 1. Introduction

Obstructive sleep apnea (OSA) is a sleep-related breathing disorder characterized by repeated partial or complete obstruction of the upper airway, leading to hypopneas or apneas [1]. Patients frequently suffer from excessive daytime sleepiness, fatigue, tiredness, snoring, gasping, and morning headaches [2]. Risk factors for OSA mainly include older age, male sex, obesity, smoking, alcohol use, family history of OSA, and craniofacial and upper airway morphology [3,4,5].

For decades, the preferred first-line treatment option for moderate-to-severe OSA has been nonsurgical ‘continuous positive airway pressure’ (CPAP) [6,7,8]. Another common non-invasive option for OSA treatment is the use of a mandibular advancement device (MAD) [9]. A disadvantage of CPAP and MAD is suboptimal long-term adherence. Surgical therapy provides a solution for OSA patients who have difficulties accepting lifelong treatment with CPAP or MAD. In an American Academy of Sleep Medicine clinical practice guideline, it is recommended that clinicians discuss and/or refer adult OSA patients with a body mass index (BMI) < 40 kg/m^2^ who are intolerant or unaccepting of positive airway pressure (PAP) to a sleep surgeon for an alternative treatment options, as part of a patient-oriented solution [10].

Maxillomandibular advancement surgery (MMA) has proven to be the most effective surgical treatment for OSA—aside from tracheostomy—with a success rate of approximately 85% [8,11,12,13]. The surgical procedure consists of a combination of a Le Fort I osteotomy for the maxilla and a bilateral sagittal split osteotomy (BSSO) for the mandible. The maxilla and mandible are both significantly advanced and rotated counter-clockwise to enlarge the upper airway’s volume and reduce upper airway soft tissue collapsibility [13,14,15]. Virtual surgical planning (VSP) is used for preoperative simulation of the MMA, and 3D-printed surgical splints are generated from the VSP to transfer the plan to the surgical setting [16]. Since large maxillomandibular complex advancement and counter-clockwise rotation contribute to a decrease in the apnea hypopnea index (AHI) and therefore treatment success, achieving these planned movements accurately during surgery is essential [12,17]. Previous research has shown that the planned surgical movements are often not accurately achieved in standard orthognathic surgery, especially in cases with larger movements [18]. Given the extensive movements involved in MMA, it is reasonable to expect that the planned movements in MMA might be even less accurately achieved compared to standard orthognathic surgery. Surprisingly, no prior studies have investigated the extent to which planned—specifically sagittal—movements are accurately achieved in MMA procedures.

The primary aim of this study was to investigate the extent to which planned advancement and counter-clockwise rotation, the two most relevant movements for surgical success in MMA for OSA surgery, are accurately achieved. The secondary aim of this study was to investigate the correlation between realized maxillary and mandibular advancement and relative AHI reduction.

## 2. Materials and Methods

### 2.1. Study Participants

Patients treated for OSA with MMA in the Department of Oral and Maxillofacial Surgery of the Amsterdam University Medical Centers (UMC) between November 2017 and March 2020 were considered for inclusion in this study. The inclusion criteria were: (1) adults aged 18 years or older; (2) diagnosed with OSA through polysomnography (PSG); (3) CPAP therapy failure or intolerance; (4) PSG conducted at least 3 months postoperatively; (5) preoperative three-dimensional (3D) virtual surgical planning of MMA; and (6) availability of a spiral computed tomography (CT) or cone-beam computer tomography (CBCT) scan after surgery. Exclusion criteria were: (1) patients undergoing other additional procedures during MMA (e.g., multi-piece Le Fort osteotomy, temporomandibular joint (TMJ) reconstruction); (2) previous Le Fort I osteotomy or BSSO; (3) cleft palate or syndromic patients; and (4) insufficient image quality for postoperative analysis. The study design was a retrospective cohort study.

This study was conducted and performed in accordance with the Declaration of Helsinki guidelines for human research. Patients were sent a letter to inform them that their medical records, polysomnography results, and radiological images were anonymously going to be used for study purposes. The option was provided to opt out of inclusion in the study. Included patients’ medical records were reviewed and data were collected. Preoperative (baseline) patient characteristics included gender, age, and body mass index (BMI).

The Medical Ethics Committee of the Amsterdam UMC decided that this study was waived for the Medical Research Human Subjects Act (W22_042 # 22.07).

### 2.2. Imaging Protocol

CT (Somatom Force, Siemens Medical Solutions, Erlangen, Germany) or CBCT (Planmeca Promax, Planmeca OY, Helsinki, Finland) scans were acquired 1 to 6 weeks preoperatively using a standardized protocol (120 kV, 300 mAs, field of view (FOV) 240 mm, pitch 0.55, slice thickness 1.0 mm, image matrix 512 × 512, window W1600/L400, hard-tissue kernel (Hr64)) or CBCT scan (84–96 kV, 100 mAs, FOV 230 mm × 170 mm (diameter × height), slice thickness 0.4 mm, image matrix 575 × 575, window/level 2500/596, pixel size 0.4 mm). Scanned patients were instructed to remain still, relax, and place the bite in a retruded contact position.

Baseline two-dimensional skeletal patterns and relationships were obtained on lateral cephalometric radiographs between 1 and 6 weeks preoperatively. Steiner radiographic cephalometric analyses were performed in Viewbox (version 4; dHAL Software, Kifissia, Greece).

### 2.3. Virtual Surgical Planning

**Figure 1:**
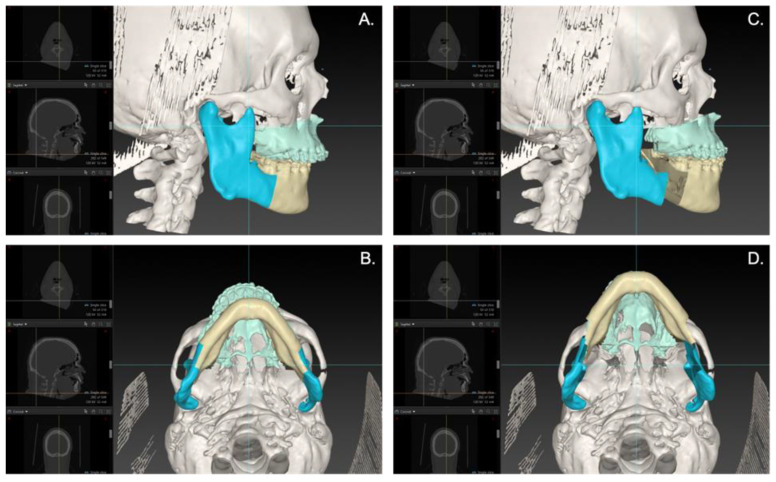
An example of a virtual plan of an MMA case. Lateral (**A**) and caudal (**B**) view of the preoperative 3D virtual hard-tissue skull model of the patient in IPS (KLS Martin, Tuttlingen, Germany). Lateral (**C**) and caudal (**D**) view of the postoperative 3D virtual hard-tissue skull model, where the maxilla and mandible are virtually osteotomized according to a Le Fort I osteotomy and BSSO. The maxilla and mandible are advanced and counter-clockwise pitched.

Preoperative CT or CBCT data were exported in digital imaging and communications in medicine (DICOM) format and imported into the Maxilim software (Medicim NV, Mechelen, Belgium) (until April 2017) or IPS CaseDesigner (KLS Martin, Tuttlingen, Germany) (from May 2017 onwards). A 3D virtual patient model was reconstructed and aligned with the patient’s natural head position (NHP) based on clinical assessment and standardized patient photos [19]. The maxilla and mandible were virtually osteotomized according to a Le Fort I osteotomy and BSSO, respectively (Figure 1). Based on the planned maxillary and mandibular position, intermediate and final splints were designed and 3D-printed for either maxilla-first or mandible-first treatment sequence based on the surgeon’s preference.

### 2.4. Surgical Technique

#### 2.4.1. Le Fort I Osteotomy

A gingivobuccal incision was made, apical, from the first molar on the right to the first molar on the left. Subperiosteal dissection and elevation of the oral soft tissue and nasal mucosa were performed. A Le Fort I osteotomy was performed using a reciprocating saw from the pterygoid processes towards the piriform rims. A glabella reference marker was placed. Down-fracturing and mobilization of the maxilla was performed with a bone hook and Rowe’s forceps. A surgical splint was used to position the maxilla in the intended planned position after the removal of interferences. Temporary maxillomandibular fixation was performed using power chains or steel wire ligatures. Rigid fixation was applied with an array of titanium miniplates and monocortical screws. Wound closure followed with absorbable sutures.

#### 2.4.2. Bilateral Sagittal Split Osteotomy (BSSO)

A mucosal incision was made with subperiosteal dissection and elevation of the oral soft tissue along the anterior border on one side of the ramus and continued inferiorly, along the external oblique ridge. A horizontal, oblique, and vertical osteotomy was placed with either a burr or reciprocating saw according to the Hunsuck modification of the Obwegeser and Dal Pont BSSO technique [20]. The bone segments were separated with osteotomes and a bone spreader. The same procedure was applied on the contralateral side. A surgical splint was used to position the mandible in the planned position, and rigid fixation was applied with an array of titanium miniplates and monocortical or bicortical screws after putting the maxillomandibular complex into temporary maxillomandibular fixation with power chains or steel wire ligatures. Wound closure followed with absorbable sutures.

In the maxilla-first surgical protocol, the Le Fort I osteotomy was performed before the BSSO, and in the mandible-first protocol, the BSSO was performed before the Le Fort I osteotomy. Antibiotics (Augmentin, GlaxoSmithKline BV, Zeist, The Netherlands) were administered at the start of the procedure and continued for 7 days postoperatively. All patients were monitored for at least one night in the intensive care or medium care unit [21].

### 2.5. Outcome Evaluation

**Figure 2:**
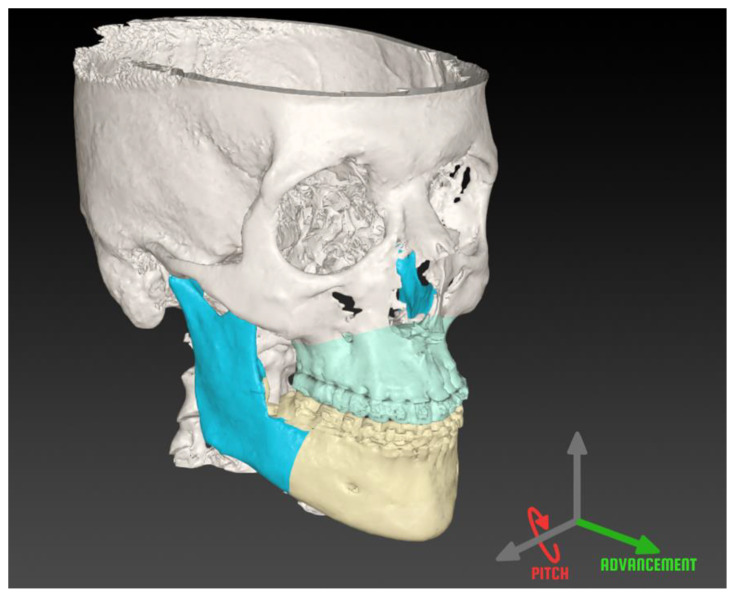
Maxillomandibular complex positioning frame. The back-to-front (advancement) translation is shown in green and pitch rotation is shown in red around the reference axes.

In order to evaluate the accuracy of the achieved postoperative result, the preoperative and postoperative DICOM data were imported into 3D MedX (3D Lab Radboudumc, Nijmegen, the Netherlands) to assess the surgical result with the OrthoGnathicAnalyser workflow [18,22]. This is a validated evaluation tool, which is able to calculate the transformation between the planned and achieved maxilla and mandible and express the deviation in clinically relevant parameters: (1) front–back translation (posteroanterior axis); (2) right–left translation (lateromedial axis); (3) up–down translation (superoinferior axis); (4) roll; (5) pitch; and (6) yaw (Figure 2). The main goal in MMA surgery is to adequately advance the maxilla and mandible and rotate them counter-clockwise in order to enlarge the upper airway. It is therefore essential to achieve the planned advancement and counter-clockwise rotation; thus, these were the parameters that were investigated in this study.

Patients received a full-night level 1 (in lab) or 2 (at home) PSG prior to MMA surgery and at least 3 months after surgery (Somnoscreen; SOMNOmedics GmbH, Randersacker, Germany). To assess sleep stages, EEG (F3, F4, C3, C4, M1, M2, O1, O2), EOG, and submental EMG were used. Nasal airflow was measured with a cannula/pressure transducer. Oronasal thermal flow determined airflow and mouth breathing. Arterial oxyhemoglobin was monitored via pulse oximetry. Thoracoabdominal excursions were measured qualitatively with respiratory belts. A position sensor determined body position, and limb movements were detected with tibial EMG. Cardiac events were scored via ECG, and snoring was recorded with a snore sensor. A clinical neurophysiologist specialized in scoring sleep studies interpreted and scored the sleep studies based on the updated 2007 criteria from the American Academy of Sleep Medicine [23]. Included PSG parameters consisted of the preoperative and postoperative apnea hypopnea index (AHI), 3% oxygen desaturation index (ODI), and lowest oxygen saturation (LSAT). According to Sher’s criteria, surgical response was defined as “at least 50% AHI reduction following MMA and a postoperative AHI < 20” [24].

### 2.6. Sample Size

Due to the nature of retrospective design, the sample size was not estimated prior to the study. A post hoc power analysis was performed for the primary outcome variables (i.e., observed differences between planned and achieved movements) using G*Power (Version 3.1.9.6, Heinrich-Heine-Universität Düsseldorf, Düsseldorf, Germany).

### 2.7. Statistical Analysis

Statistical analysis was performed using SPSS (version 29.0; IBM Corp., Armonk, NY, USA) and R (R Development Core Team, Vienna, Austria). Descriptive statistics were calculated for all demographic and outcome variables. Mean, standard deviation, median, interquartile range (IQR), and/or range were used to report the continuous variables, and frequency and percentage were used for summarizing categorical variables. Normality was tested using the Shapiro–Wilk test. To compare the paired continuous values, the paired-samples t-test (for data with a normal distribution) or Wilcoxon’s signed-rank test (for non-normal data) were used. To compare continuous values between the maxilla-first and mandible-first surgical sequence group, the independent-samples t-test was used when data were normally distributed, and the Mann–Whitney U test was used when data were not normally distributed. Linear regression analysis was performed to investigate the association between the planned movement and the difference between the planned and achieved movement. Adjusted R-squared (R^2^) value was used to quantify the proportion of the variance that could be explained by the planned movement in the linear regression model. The relative AHI improvement was calculated, and a Pearson correlation analysis was used to investigate its relationship with the amount of maxillary and mandibular advancement. For all analyses, a *p*-value < 0.05 was considered statistically significant.

## 3. Results

### 3.1. Study Participants

In total, 27 patients underwent MMA for OSA in the Department of Oral and Maxillofacial Surgery, Amsterdam UMC, between November 2017 and March 2020. One patient opted out of the study, and ten patients were excluded due to the fact that the 3D-imaging protocol was not followed correctly, which was mostly due to the absence of a CT or CBCT scan after surgery (*n* = 7). Therefore, 16 patients were included in this study; 10 were male and 6 were female. The mean age was 53 ± 9 years (range 36–69 years) (Table 1). Among the included sixteen patients, all patients (100%) had treatment failure or intolerance to CPAP, twelve patients (75%) had treatment failure or intolerance to MAD, and seven patients (44%) had other type(s) of upper airway surgery prior to MMA.

### 3.2. Planned vs. Realized Movements

**Figure 3:**
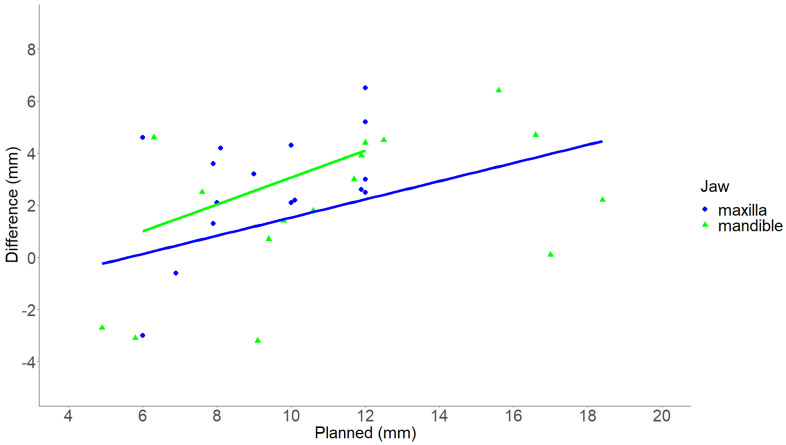
Scatter plot illustrating the relationship between the planned advancement and the difference between planned and realized advancement. The X-axis illustrates the planned advancement in mm. The Y-axis illustrates the difference between planned and realized advancement in mm. Each green triangle depicts an individual maxilla, and each blue dot depicts an individual mandible. The green and blue lines illustrate the linear regression of the maxilla and mandible data, respectively. Mm, millimeters. *p*-value < 0.05 was considered statistically significant.

**Figure 4:**
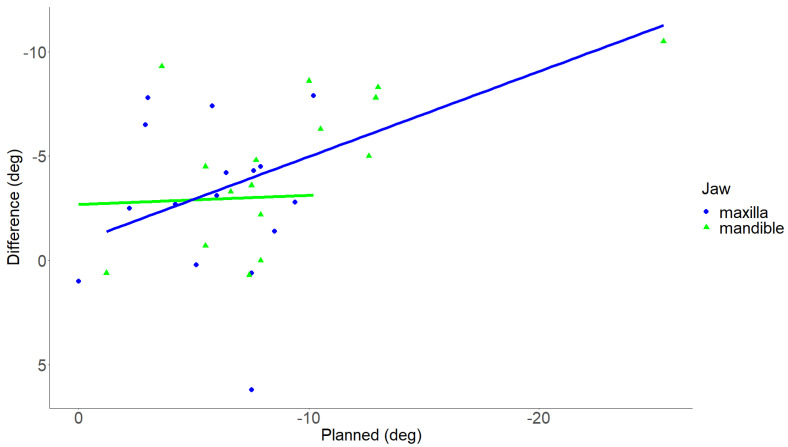
Scatter plot illustrating the relationship between the planned counter-clockwise rotation and the difference between planned and realized counter-clockwise rotation. The X-axis illustrates the planned pitch in deg. The Y-axis illustrates the difference between planned and realized counter-clockwise rotation in deg. Each green triangle depicts an individual maxilla, and each blue dot depicts an individual mandible. The green and blue lines illustrate the linear regression of the maxilla and mandible data, respectively. Deg, degree. *p*-value < 0.05 was considered statistically significant.

The median planned advancement of the maxilla was 9.5 mm (range 6.0–12.0 mm), and the median planned advancement for the mandible was 11.2 mm (range 4.9–18.4 mm). The planned median counter-clockwise rotation for the maxilla and mandible were 6.2° (range 0.0–10.2°) and 7.8° (range 1.2–25.4°), respectively. For both the maxilla and mandible, the achieved advancement and counter-clockwise rotation were significantly smaller than the planned advancement and rotation (*p* < 0.05) (Table 2). The study revealed that a larger advancement corresponded to a larger difference between the planned and realized advancement for both the maxilla and mandible. Notably, this difference was only found to be statistically significant for the maxilla (*p* = 0.048; adjusted R^2^ = 0.20) and not for the mandible (*p* = 0.06; adjusted R^2^ = 0.18) (Figure 3). A larger counter-clockwise rotation was associated with a significantly greater difference between the planned and realized counter-clockwise rotation for the mandible (*p* = 0.012; adjusted R^2^ = 0.33) but not for the maxilla (*p* = 0.9; adjusted R^2^ = 0.07) (Figure 4).

### 3.3. Maxilla-First Surgical Sequence vs. Mandible-First Treatment Sequence

In the comparative analysis between the maxilla-first surgical sequence and the mandible-first treatment sequence, Table 3 serves to demonstrate their collective inability to achieve the intended movements accurately. In the analysis, the discrepancies between the planned and achieved movements between the maxilla-first and mandible-first surgical sequences were all not statistically significantly (*p* > 0.45).

### 3.4. Amount of Advancement and the Relative AHI Improvement

The median AHI was significantly reduced from 62.6 (6.4–84.0) events/h to 19.4 (3.9–47.0) events/h (*p* = 0.001). Overall success was achieved in 63% of the cases (Table 4).

**Figure 5:**
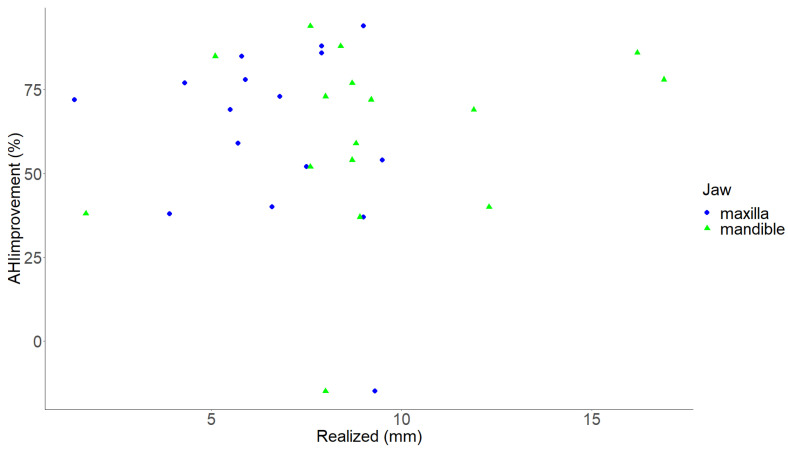
Scatter plot illustrating the relationship between the realized maxillary and mandibular advancement and the percentage of AHI improvement. The X-axis illustrates the realized advancement in mm. The Y-axis illustrates the AHI improvement in %. Each green triangle depicts an individual maxilla, and each blue dot depicts an individual mandible. AHI, apnea hypopnea index; mm, millimeters; %, percentage. *p*-value < 0.05 was considered statistically significant.

No association was found between the amount of realized maxillary and mandibular advancement and the relative AHI improvement (*p* = 0.389 and *p* = 0.387 respectively) (Figure 5).

## 4. Discussion

Previous studies have looked into the accuracy of orthognathic surgery [18,22,25,26]. However, as far as the authors are aware of, none have explored the extent to which planned surgical movements are accurately achieved in MMA procedures [17,18]. Therefore, the present study aimed to investigate the extent to which preoperative planned advancements and counter-clockwise rotations were achieved during MMA surgery for OSA patients.

One of the main findings of this preliminary study is the consistent trend of underachievement of the desired advancements in the MMA cases. This is well in line with findings in traditional orthognathic surgery for the correction of dentofacial discrepancies [22]. Notably, these discrepancies may be attributed to various factors, for example altered seated position of the condyle as a result of different muscular tone and patient positioning intraoperatively [22,27].

In addition to the difference found between the planned and realized advancement, the results also show that the realized counter-clockwise rotation for both the maxilla and mandible were consistently less than planned. Liebregts et al. found a similar difference between the planned and realized counter-clockwise rotation in bimaxillary osteotomies in traditional orthognathic surgery [18]. The possible reasons for this might be positioning errors intraoperatively due to interfering bone segments between the osteotomized maxilla and the pterygoid plates or a non-centric relation of the mandible during temporary maxillomandibular fixation with the use of intraoperative surgical splints [18,22].

Both findings emphasize that although virtual surgical planning and CAD/CAM intraoperative surgical splints are utilized for MMA nowadays, it is still difficult to accurately achieve the planned advancement and counter-clockwise rotation. The relatively large surgical splints that are frequently used in MMA surgery, due to the large planned displacements, might be a significant factor in decreasing surgical accuracy. This might explain the results of this study, which showed that the surgical accuracy was further reduced when the planned advancements and counter-clockwise rotations increased. The paper by Liebregts et al. and Stokbro et al. also alluded to this finding [18,28].

The mandible-first sequence has been proposed as a solution to address issues with centric relation and, consequently, to enhance the predictability of achieving the intended position [29]. However, no significant beneficial effect could be demonstrated in this small sample size. The choice between the maxilla-first and mandible-first surgical sequences is often influenced by surgeon preferences [30,31]. In cases of OSA, concerns about achieving the desired maxillary advancement due to limitations in soft tissue (e.g., through scarring due to previous upper airway surgery) might be present. In our hospital, a strategic approach is often used that is only possible within the maxilla-first sequence. It involves the use of two intermediate splints: one for larger advancement (e.g., 12 mm) and another for a slightly lesser advancement (e.g., 10 mm) as a precautionary ‘back-up’.

Multiple studies have reported the association between MMA success and the amount of advancement [12,17], but others have reported no association between the amount of planned advancement and AHI improvement after MMA [32,33]. A possible explanation for these inconsistent findings could be that the planned advancement instead of the realized advancement has been used, or because there is variation in the use of two-dimensional and three-dimensional imaging methods [17,34,35].

As a secondary objective, this study investigated the correlation between the realized advancement and the relative AHI reduction, but no significant correlation was found. This lack of correlation may be attributed to the low number of patients included and the extensive complexity of OSA, where treatment success or improvement depends on various interacting factors, including demographic characteristics, anatomical hard-tissue and soft-tissue parameters, PSG specifics, and surgical characteristics [35,36,37,38,39]. This finding raises the question of whether or not more accurate achievement of the planned advancement and counter-clockwise rotation is actually necessary through, for example, a splintless surgical workflow [40,41,42,43]. This is especially true when looking at the finding that the median AHI was significantly reduced from approximately 63 events/h to 19 events/h despite consistently not achieving the planned displacements. Some cases still showed a significant relative AHI reduction despite a small advancement, as seen in Figure 5.

The amount of advancement and counter-clockwise rotation necessary for surgical success remains unknown. A major advantage of the present study is the fact that a validated workflow and tool, the OrthoGnathicAnalyser, was used in order to measure the discrepancy between the planned and the realized result in three dimensions with the use of CT and CBCT. The argument could be raised that an error distribution between the CT- and CBCT-based registration could have influenced the outcome of this study. However, based on the validated findings of Eggers et al. this can be considered as negligible [44]. Although the OrthoGnathicAnalyser tool is able to accurately measure all translational and rotational movements, the main focus in this study was on the advancement and the counter-clockwise rotation of the maxilla and the mandible, as these are essential factors contributing to the relief of patients’ OSA [14,45,46,47,48]. However, caution is warranted in interpreting the results because of the study limitations (small population, potential biases in retrospective design, and low inclusion rate). In the present study, the powers of the primary outcomes (i.e., maxillary advancement, mandibular advancement, maxillary counter-clockwise rotation, and mandibular counter-clockwise rotation) were 1, 0.7, 0.8, and 1, respectively. This indicated that except for mandibular advancement, all other primary outcome variables had sufficient power in the statistical analyses. It is recommended that future studies—preferably prospective studies in large cohorts—should be undertaken to verify the current findings, especially since the literature on the topic is scarce. Additional future research should further investigate which factors in MMA surgery contribute most to surgical success and to optimize surgical planning for individual patients.

## 5. Conclusions

This study emphasizes the importance of acknowledging the presence of surgical inaccuracies in MMA procedures for patients with OSA and underscores the need for heightened awareness among surgeons and future research endeavors. Furthermore, our findings propose that the extent of maxillomandibular complex advancement may not hold paramount significance in determining the outcome of MMA treatment. Therefore, further investigations and refinements in surgical techniques are imperative to optimize the efficacy of MMA procedures.

## Figures and Tables

**Table 1:** Baseline characteristics of the study population.

Total Population (*n* = 16)		Mean ± SD	Range
Male (*n* (%))	10 (62.5)		
Age (years)		52.9 ± 9.3	36–69
BMI (kg/m^2^)		27.1 ± 3.9	18.0–32.4
∠SNA (degrees)		80.40 ± 3.9	69.6–88.1
∠SNB (degrees)		73.9 ± 7.4	52.0–83.8
∠ANB (degrees)		6.1 ± 5.0	−0.1–17.7
∠OP-SN (degrees)		20.8 ± 12.0	5.0–59.9
∠MP-SN (degrees)		42.5 ± 16.3	13.6–90.5

Gender is presented as number of patients and percentage. Age, BMI, ∠SNA, ∠SNB, ∠ANB, ∠OP-SN, and ∠MP-SN are presented in years, kg/m^2^, and degrees. ∠ANB, angle between the A/nasion plane and the nasion/B plane; BMI, body mass index; cm, centimeters; kg/m^2^, kilograms per square meter; ∠MP-SN, angle between the mandibular plane and the sella/nasion plane; ∠OP-SN, angle between the occlusal plane and the sella/nasion plane; SD, standard deviation; ∠SNA, angle between the sella/nasion plane and the nasion/A plane; ∠SNB, angle between the sella/nasion plane and the nasion/B plane. *p*-value < 0.05 was considered statistically significant.

**Table 2:** Comparison between planned and achieved advancement (B-F translation) and counter-clockwise rotation (anticlockwise pitch) for maxilla and mandible.

		Planned			Achieved			Difference			*p*-Value
		Median	IQR	Range	Median	IQR	Range	Median	IQR	Range	
Counter-clockwise rotation (degrees)	Maxilla	6.2	3.9–7.7	0.0–10.2	2.6	0.7–5.6	4.8–13.7	3.7	1.7–6.2	−6.1–7.9	0.006
	Mandible	7.8	6.3–11.0	1.2–25.4	4.5	2.6–6.2	−5.7–14.9	4.7	1.2–8.2	−0.8–10.5	0.001
Advancement (mm)	Maxilla	9.5	7.9–11.9	6.0–12.0	6.7	5.7–8.2	1.4–9.5	3.1	2.1–4.3	0.6–12.0	0.002
	Mandible	11.2	8.7–13.3	4.9–18.4	8.7	7.9–9.9	1.7–16.9	2.3	0.3–4.6	−3.1–6.4	0.03

Translations are presented in mm. Rotations are presented in degrees. B-F translation, translation from back to front; mm, millimeters; IQR, interquartile range = quartile 3—quartile 1. *p*-value < 0.05 was considered statistically significant.

**Table 3:** Comparison of discrepancies between planned and achieved sagittal movements in maxilla-first vs. mandible-first surgical sequences.

		Maxilla-First (*n* = 7)			Mandible-First (*n* = 9)			*p*-Value
		Median	IQR	Range	Median	IQR	Range	
Counter-clockwise rotation (degrees)	Maxilla	4.3	2.5–6.5	0.3–7.8	2.8	1.2–6.8	0.6–7.9	0.71
	Mandible	4.5	3.3–8.7	0.8–9.2	4.8	0.7–7.3	0.0–10.5	0.50
Advancement (mm)	Maxilla	3.1	2.1–4.3	2.1–5.1	2.6	1.7–4.1	0.6–6.5	0.66
	Mandible	1.8	1.4–4.6	0.1–4.6	3.1	2.4– 4.3	0.7–6.4	0.45

Rotations are presented in degrees. Translations are presented in mm. B-F translation, translation from back to front; mm, millimeters; IQR, interquartile range = quartile 3—quartile 1. *p*-value < 0.05 was considered statistically significant.

**Table 4:** PSG results before and after MMA for total population.

	Total Population (*n* = 16)				
	Mean	SD	Median	IQR	Range
Pre-op AHI (events/h)	49.8	23.8	62.6	43.5–77.7	6.4–84.0
Post-op AHI (events/h)	17.3	12.8	19.4	10.8–29.9	3.9–47.0
Pre-op ODI (events/h)	50.9	25.8	64.7	45.6–75.8	2.2–93.4
Post-op ODI (events/h)	21.0	13.9	19.5	10.5–31.8	3.0–51.1
Pre-op LSAT (%)	74.7	12.0	76	63–80	52–92
Post-op LSAT (%)	82.4	8.8	85	75–87	64–92
Success (%)	10/16 (62.5)				
Cure (%)	3/16 (18.8)				

PSG results are presented as events/hour or percentage. Success and cure are presented as percentage. AHI; apnea hypopnea index; events/h, post-op, after MMA; pre-op, prior to MMA; N, number of patients; LSAT, lowest oxygen saturation; MMA, maxillomandibular advancement; ODI, oxygen desaturation index; PSG, polysomnography; IQR, interquartile range = quartile 3—quartile 1; SD, standard deviation. *p*-value < 0.05 was considered statistically significant.

## Data Availability

The data presented in this study are available on request from the corresponding author.
